# A comparative study of nebivolol and (S) atenolol on blood pressure and heart rate on essential hypertensive patients

**DOI:** 10.4103/0253-7613.71918

**Published:** 2010-12

**Authors:** G.N. Sahana, N. Sarala, T.N. Kumar, V. Lakshmaiah

**Affiliations:** Department of Pharmacology, India; Department of Medicine, Sri Devaraj URS Medical College and R. L. Jalappa Hospital and Research Center, Tamaka Kolar-563 101, India

**Keywords:** Essential hypertension, nebivolol, (S)-atenolol

## Abstract

**Objectives:**

To study the effect of nebivolol 5 mg once daily versus (S)-atenolol 25 mg once daily in patients with essential hypertension.

**Materials and Methods:**

A prospective study was conducted at RLJH and Research Centre which included 30 patients in each group with essential hypertension. The sex, age, presenting illness, and family history of the patients were recorded. Investigations such as blood sugar, urine analysis, kidney function test, lipid profile, and ECG were performed before starting the treatment. Any adverse effects during the treatment were noted. Blood pressure and heart rate were recorded at baseline and during follow-up. One group received nebivolol 5 mg once daily and other group (S)atenolol 25 mg once daily. Patients were followed-up every 15 days for 3 months.

**Results:**

Nebivolol group had 18 males and 12 females with mean age 50.6 ± 9.5 years, (S)-atenolol had 16 males and 14 females with mean age 54.4 ± 9 years. Patients receiving nebivolol and (S)-atenolol showed a significant fall (*P* <·0001) in systolic (SBP), diastolic blood pressure (DBP), and heart rate at the end of first, second, and third month when compared to baseline. The difference in fall in SBP and DBP was insignificant between the groups, but fall in heart rate was significant (*P* <·0001). Adverse effects such as headache, dizziness, and fatigue were reported with both drugs.

**Conclusion:**

Reduction of blood pressure with nebivolol and (S)atenolol was similar, but fall in blood pressure from baseline was highly significant in both groups.

## Introduction

Hypertension is a major public health problem, being one of the leading causes of death and disability worldwide and a major risk factor for cardiovascular diseases.[1–3] The poor control of hypertension appears to be the result of poor detection and awareness of the disease coupled with inadequate treatment. The lowering of elevated blood pressure reduces morbidity from stroke, myocardial infarction, congestive cardiac failure, and renal failure.[4]

Essential hypertension is a condition where the cause for rise in blood pressure is not known.[5] It is sometimes associated with endothelial dysfunction which is caused by production of oxygen free radicals that destroy nitric oxide (NO) and impair its beneficial and protective effects on vessel wall. The endothelial dysfunction is thus a mechanism promoting atherosclerosis and thrombosis, contributing to cardiovascular events. It is now considered as an important target for cardiovascular treatment.[6]

Beta-blockers are widely accepted as the first-line treatment for hypertension.[7,8] Nebivolol is a selective β_1_-lipophilic blocker,[9] it also modulates NO release. (*S*)-atenolol is a hydrophilic β _1_-blocker which is an optically pure enantiomer of atenolol.[10] Fifty milligrams of (*S*)-atenolol is equivalent to 100 mg of racemic atenolol in its efficacy.[11] To the best of our knowledge, there are no studies available comparing the effect of nebivolol and (*S*)-atenolol on blood pressure and heart rate in patients with essential hypertension, hence this study was done.

## Materials and Methods

A prospective study was conducted from 01.04.2004 to 31.03.2005 in patients with essential hypertension. Sixty patients were included in the study, of which 30 patients received nebivolol and 30 received (*S*)-atenolol. The study was conducted in the Medicine and Cardiology Department of R. L. Jalappa Hospital and Research Centre, attached to Sri Devaraj Urs Medical College, Tamaka, Kolar.

A proforma containing detailed information on each patient was prepared according to the protocol designed for the study. Ethics clearance was obtained from Institutional Ethics Committee. Informed consent was taken from all the patients included in the study. Patients of either sex in the age group of 30–80 years with blood pressure of ≥140/90 mmHg were included in the study. The upper limit of blood pressure in both groups was 180/110 mmHg. Patients belonging to both stage 1 and stage 2 hypertension were selected as per JNC VIIth report. Patients with secondary hypertension, diabetes mellitus, allergic to nebivolol, and (*S*)-atenolol, suffering from asthma or liver dysfunction, heart block, peripheral artery disease, pregnant, and lactating women and those who had received antihypertensive treatment were excluded from the study. The patients above 65 years had associated diseases such as diabetes mellitus, asthma, and liver dysfunction, so they were excluded from the study. Only newly diagnosed hypertensive patients without prior antihypertensive treatment and without any associated diseases mentioned earlier were included.

Blood pressure and heart rate were measured in sitting posture using the standard mercury sphygmomanometer. Blood pressure was recorded after giving rest of 15 min to patients in sitting position. Two recordings of blood pressure were taken at an interval of 5 min. Before administering the drug, the baseline blood pressure and heart rate were recorded. Nebivolol 5 mg once daily and (*S*)-atenolol 25 mg once daily were given to respective patients in each group. The patients were advised to report for follow-up every 15 days for 3 months. On each visit, blood pressure and heart rate were recorded. Blood sugar, urine analysis, renal function test, lipid profile, and ECG were assessed before starting the treatment. Lipid profile was repeated at the end of 3 months. The data obtained were analyzed using descriptive statistics and paired and unpaired Student’s *“t”* test to compare results within the group and between groups, respectively.

## Results

Sixty patients were included in the study, of which 30 received nebivolol 5 mg once daily and the other 30 patients received (*S*)-atenolol 25 mg once daily. All the patients completed the study.

Table 1 depicts the demographic data of the patients. In the nebivolol group, 60% were males and 40% females with their mean age being 50.6 ± 9.5 years. Among the patients who received (*S*)-atenolol, 53% were males and 47% females with mean age 54.4 ± 9 years. Ten male patients and five female patients of nebivolol group, nine male and nine female patients of (*S*)-atenolol group had family history of hypertension. The mean systolic blood pressure (SBP) and diastolic blood pressure (DBP) (at baseline) of patients with history of smoking and alcoholism in both the groups were the same as that of other patients included in the study. Therefore, these patients were included in the study. The most common presenting symptoms in both the groups were headache and giddiness. Seven patients in each group presented with epistaxis. These patients had blood pressure in the range of 140/90 to 160/100 mmHg, and they had no other bleeding disorders.

**Table 1:** Demographic characteristics of nebivolol and (*S*)-atenolol group

*Particulars of the patients*	*Nebivolol*	*(S)-Atenolol*
Number	30	30
Mean age (years)	50.6 ± 9.5	54.4 ± 9
Sex (M/F)	18/12 (60/40)	16/14 (53/47)
Alcohol consumption	13 (43)	10 (33)
History of smoking	12 (40)	8 (27)
Family history of hypertension	15 (50)	18 (60)

Mean ± SD, Number in parentheses represents percentage.

The patients were followed up at 15 days, but when the data were analyzed at the interval of 15 days the results were statistically insignificant. Therefore, the data were assessed at monthly intervals.

**Figure 1:**
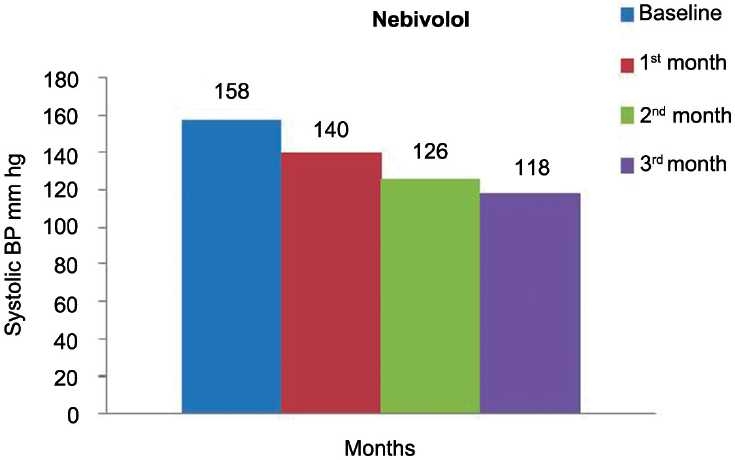
Effect of nebivolol on systolic blood pressure.

**Figure 2:**
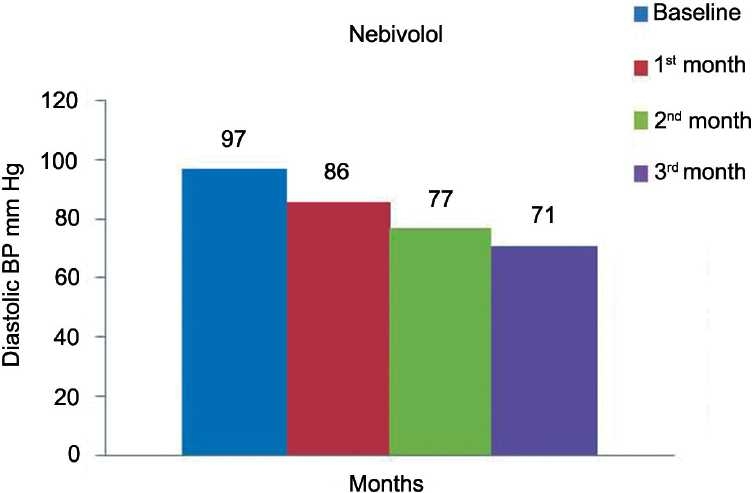
Effect of nebivolol on diastolic blood pressure.

**Figure 3:**
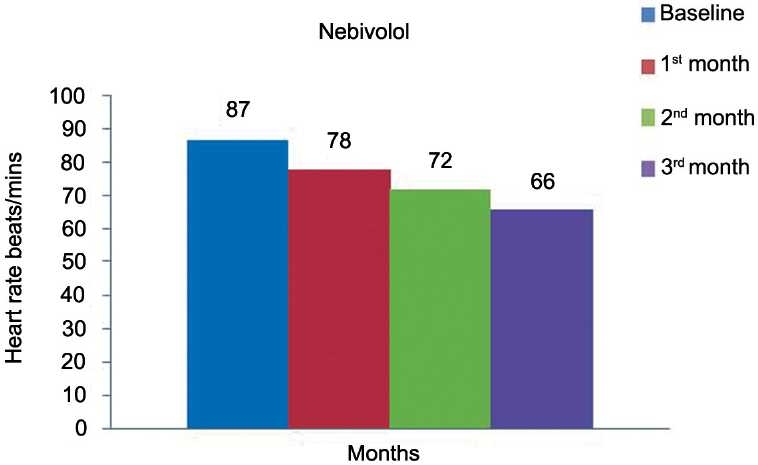
Effect of nebivolol on heart rate.

Table 2 and Figures 1–3 show the effect of nebivolol on SBP, DBP, and heart rate at the end of 1, 2, and 3 months. The values in Table 2 are represented as mean ± SD. There was a significant fall (*P* < 0·0001) in SBP at the end of each month (140 ± 16, 126 ± 13, 118 ± 8) as compared to the baseline (158 ± 17 mmHg) [Table 2], respectively. As depicted in Table 2, there was a significant (*P* < 0·0001) fall in diastolic blood pressure (DBP) at the end of 1, 2, and 3 months as compared to the baseline. There was gross reduction in heart rate at the end of first, second, and third months as compared to the baseline [Table 2].

**Table 2:** Effect of nebivolol and (S)-atenolol on systolic and diastolic blood pressures and heart rate

	*Nebivolol-treated group*				*(S)–Atenolol-treated group*			
	*Baseline*	*1 month*	*2 months*	*3 months*	*Baseline*	*1 month*	*2 months*	*3 months*
SBP, mmHg	158 ± 17*	140 ± 16*	126 ± 13*	118 ± 8*	160 ± 16#	142 ± 15#	131 ± 13#	115 ±7#
DBP, mmHg	97 ± 10*	86 ± 8*	77 ± 5*	71 ± 3*	99 ± 10#	89 ± 8#	79 ± 6#	71 ± 3#
HR, beats/min	87 ± 10*	78 ± 8*	72 ± 7*	$66 ± 5*	85 ± 5#	73 ± 5#	66 ± 4#	$61 ± 2#

SBP, Systolic blood pressure; DBP, diastolic blood pressure; HR, heart rate, mean ± SD

* *P* <·0001 compared with baseline

# *P* <·0001 compared with baseline

$ *P* <·0001 compared between nebivolol and (*S*)—atenolol; Statistical test used is descriptive statistics and paired and unpaired Student’s *‘t’*-test to compare results within the group and between groups, respectively.

**Figure 4:**
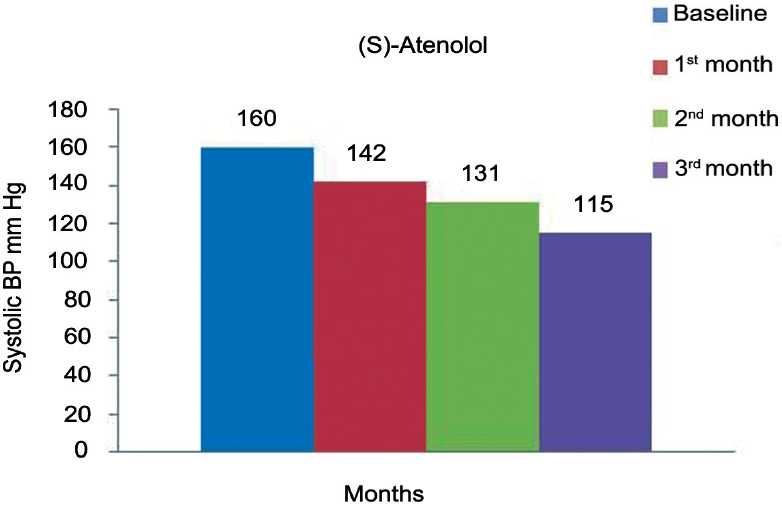
Effect of (*S*)-atenolol on systolic blood pressure.

**Figure 5:**
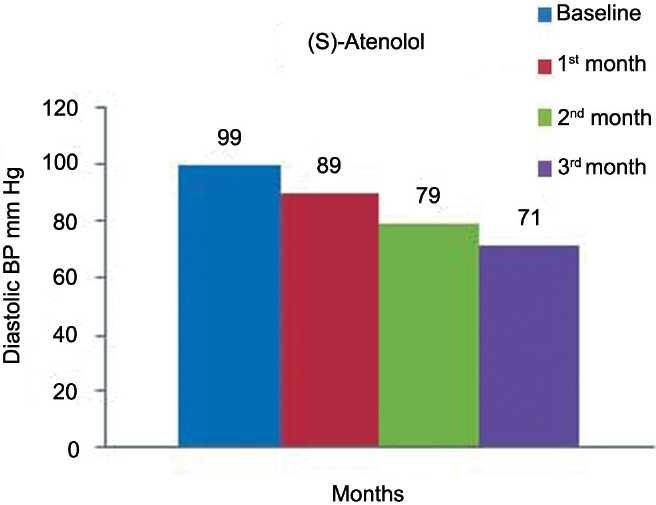
Effect of (*S*)-atenolol on diastolic blood pressure.

**Figure 6:**
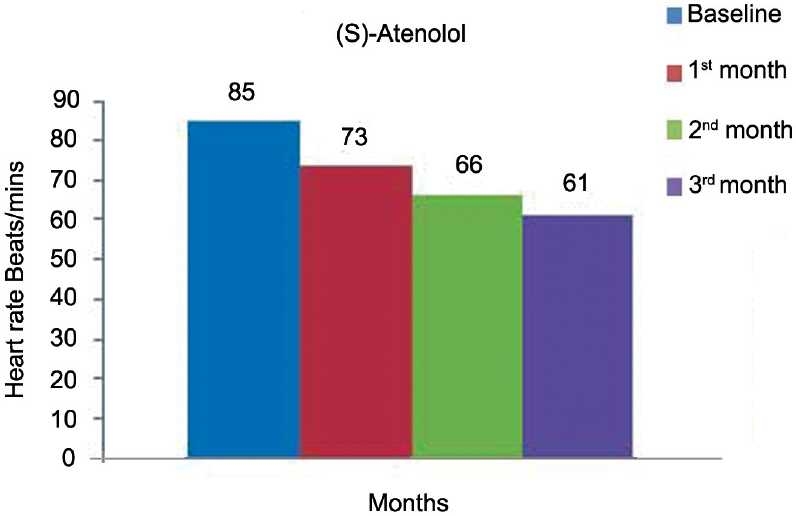
Effect of (*S*)-atenolol on heart rate.

Table 2 and Figure 4 represent the effects of (*S*)-atenolol on SBP. The mean SBP at the baseline was 160 ± 16 mmHg was reduced to 142 ± 15, 131 ± 13, 115 ±7 mmHg at the end of 1, 2, and 3 months of treatment, respectively, (*P* < 0·0001). Mean DBP as shown Table 2 and Figure 5 was decreased from 99 ± 10 mmHg to 89 ± 8, 79 ± 6, 71 ± 3 mmHg at the end of each month which is also significant (*P* < 0·0001). Table 2 and Figure 6 show the effect of (*S*)-atenolol on heart rate which was reduced from 85 ± 5 to 73 ± 5, 66 ± 4, 61 ± 2 at the end of first, second, and third month, respectively, (*P* < 0·0001).

**Figure 7:**
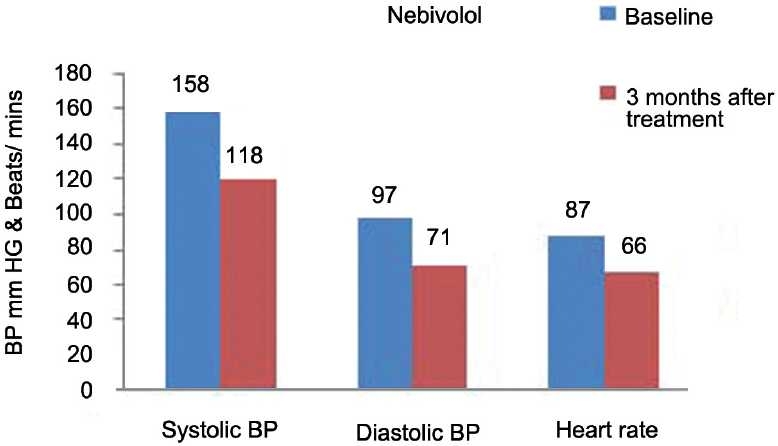
Effect of nebivolol on systolic, diastolic blood pressure, and and heart rate.

**Figure 8:**
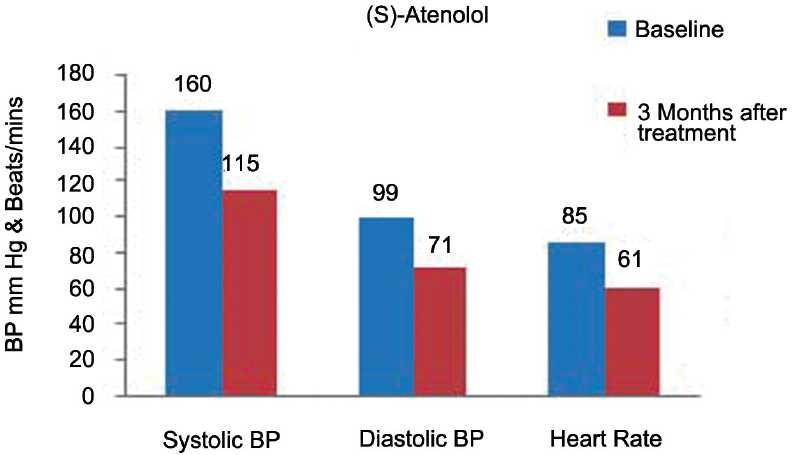
Effect of (*S*)-atenolol on systolic, diastolic blood pressure, and heart rate.

Figures 7 and 8 show the comparative effect of nebivolol and (*S*)-atenolol on SBP, DBP, and heart rate at baseline and at the end of 3 months of treatment. The mean SBP at baseline in nebivolol group was 158 ± 17 mmHg and in (*S*)-atenolol group, 160 ± 16 mmHg. At the end of 3 months, it was reduced to 118 ± 8 mmHg in nebivolol group and 115 ± 7 mmHg in (*S*)-atenolol group. The fall in blood pressure between groups after 3 months was almost similar (*P* < 0·2). Similarly, mean DBP at baseline in nebivolol and (*S*)-atenolol group was 97 ± 10 mmHg and 99 ± 10 mmHg and at the end of 3 months of treatment the fall in blood pressure in both the groups was 71 ± 3 mmHg (*P* < 0·9).

The effect of nebivolol and (S)-atenolol on heart rate is depicted in Table 2 and Figures 7 and 8. The mean heart rate at baseline was 87 ± 10 beats/min and 85 ± 5 beats/min, respectively, which was reduced to 66 ± 5 beats/min and 61 ± 2 beats/min 3 months after treatment between the groups (*P* < 0·0001).

The common adverse effects seen with both the drugs were headache, dizziness, and fatigue. Lipid profile was done as one of the laboratory tests because nebivolol has shown to decrease total cholesterol levels when compared with atenolol. There was no change in the lipid profile observed in both the groups at the end of 3 months of treatment with these drugs.

## Discussion

Hypertension remains a major health problem being one of the leading causes of death and disability. Prevalence of hypertension among Indians is 26.78% in males and 27.65% in females.[12] Cardiovascular morbidity and mortality increases as both SBP and DBP rise. Essential hypertension is associated with endothelial dysfunction, which is caused mainly by the production of oxygen free radicals that destroy NO and impair protective effects on the vessel wall. Impairment in endothelium-dependent vasodilatation[13] is known to precede atherosclerosis and particularly coronary artery disease.[14] Nebivolol, which has been used in our study has been shown in earlier studies to have the vasodilator effect by stimulating the release of potent vasodilator, NO from endothelial cells.[15–18] A possible antioxidant property of nebivolol has also been suggested as an additional factor in increasing NO bioactivity or reducing endothelin release.[19,20]

In our study, we have analyzed the effect of nebivolol 5 mg once daily and (*S*)-atenolol 25 mg once daily in hypertensive patients. The patients were in the age group of 50–54 years in both groups. Literature search has revealed comparison of effect of nebivolol 5 mg once daily with racemic mixture of atenolol 100 mg once daily on blood pressure, heart rate, and oxidative stress in essential hypertensive patients.[21] Studies comparing nebivolol 5 mg versus (*S*)-atenolol 25 mg were lacking, hence this study was carried out. In our study, we have observed a reduction of SBP and DBP and heart rate in patients who received nebivolol 5 mg once daily. The effect was observed at the end of 1 month of treatment and was maintained till the end of 3 months. These results are similar to the other studies.[21,22] In one study,[23] 100 mg of racemic mixture of atenolol (100 mg R,S-atenolol) and 50 mg (*S*)-atenolol reduced SBP as well as DBP and heart rate. In this study, (*S*)-atenolol 25 mg once daily reduced SBP, DBP, and heart rate significantly after the patients were treated for a period of 1 month and subsequently till the end of 3 months. Studies[23,24] have shown that (*S*)-enantiomer of atenolol contributes to the β-blocking activity of currently used rac-atenolol 100 mg, but the same effect was achieved with half dose (50 mg) of optically pure (*S*)-atenolol alone. In our study, we have observed that 25 mg of (*S*)-atenolol alone could reduce SBP, DBP, and HR significantly (*P* < 0·0001) at the end of 1 month of treatment and it was maintained throughout our study. Incidentally, the cost of one tablet of racemic mixture atenolol 100 mg ranges from Rs. 1.80 to 3.30 and that of 25 mg (*S*)-atenolol is Rs. 2.50.[25]

In this study both the drugs nebivolol and (*S*)-atenolol reduced SBP, DBP, and HR, but the reduction of SBP and DBP when compared between the groups at the end of 3 months was not statistically significant. Similarly, DBP was similar in both the groups. The reduction was similar in both the groups. When heart rate was compared between nebivolol-treated and (*S*)-atenolol-treated patients, there was significant fall (*P* < 0·0001) in the heart rate in (*S*)-atenolol group, this could be because (*S*)-atenolol blocks β_1_-receptors in the heart while nebivolol because of its vasodilator property can produce reflex increase in heart rate. Similar findings, i.e., reduction in SBP, DBP, and heart rate were observed in two other studies.[23,24]

The results of this study showed that nebivolol 5 mg and (*S*)-atenolol 25 mg had similar antihypertensive effect. Although there was no difference between the groups with respect to reduction of SBP and DBP, decrease in HR was significantly greater in patients receiving (*S*)-atenolol. The cost of nebivolol 5 mg is Rs. 5 per tablet and that of (*S*)-atenolol is Rs. 2.50 per tablet, thus (*S*)-atenolol being more cost-effective. The important clinical implication is that nebivolol though costly can improve nitric-oxide availability, thus prevent the endothelial dysfunction that predisposes to essential hypertension. Though this appears to offer more benefit based on its pharmacodynamics, clinically significant data were not evident in this study. Further studies with higher doses of nebivolol may throw more light on this particular aspect. Adverse effects in both the groups were similar, and no change in lipid profile was observed at the end of 3 months with either drugs.
